# Detection of Alpha-Fetoprotein Using Aptamer-Based Sensors

**DOI:** 10.3390/bios12100780

**Published:** 2022-09-21

**Authors:** Lei Liu, Huixing Wang, Bing Xie, Bianjiang Zhang, Yuanwei Lin, Li Gao

**Affiliations:** 1 Department of Kidney Transplantation, The Second Xiangya Hospital of Central South University, Changsha 410011, China; 2 School of Life Sciences, Jiangsu University, Zhenjiang 212013, China; 3 Department of Obstetrics and Gynecology, The Fourth People’s Hospital of Zhenjiang, Zhenjiang 212000, China; 4 School of Food Science, Nanjing Xiaozhuang University, Nanjing 211171, China

**Keywords:** Alpha-fetoprotein, detection, aptamer, optical biosensors, electrochemical biosensors

## Abstract

Alpha-fetoprotein (AFP) is widely-known as the most commonly used protein biomarker for liver cancer diagnosis at the early stage. Therefore, developing the highly sensitive and reliable method of AFP detection is of essential demand for practical applications. Herein, two types of aptamer-based AFP detection methods, i.e., optical and electrochemical biosensors, are reviewed in detail. The optical biosensors include Raman spectroscopy, dual-polarization interferometry, resonance light-scattering, fluorescence, and chemiluminescence. The electrochemical biosensors include cyclic voltammetry, electrochemical impedance spectroscopy, and giant magnetic impedance. Looking into the future, methods for AFP detection that are high sensitivity, long-term stability, low cost, and operation convenience will continue to be developed.

## 1. Introduction

According to data recorded, hepatocellular carcinoma (HCC) is one of the ten most common cancers in the world, and it is usually the cause of death in patients with liver cirrhosis [1]. At present, most clinical techniques, such as imaging and histology, can only play a role in advanced liver cancer [2]. The detection technology of serum tumor biomarkers can diagnose related cancers more accurately. As the most commonly used protein biomarker for liver cancer diagnosis, alpha-fetoprotein (AFP) has attracted much attention in recent years [3]. AFP is usually produced by the yolk sac of the fetus, which is a plasma protein with a molecular weight of about 70 kDa secreted from the liver, at ages between 7~8 months [4,5]. With the birth and growth of the fetus, its content gradually decreases. In the serum of healthy people, AFP concentration is less than 25 ng/mL; it is almost undetectable. However, according to statistics data, in nearly 75% of HCC patients, the AFP concentration is significantly increased to 500 ng/mL [6,7]. High levels of AFP in adult blood may indicate the presence of certain types of cancer, especially for HCC, gastric cancer, pancreatic cancer, ovarian cancer, or testicular cancer. Therefore, clinically elevated levels of AFP in adult serum are widely regarded as early indications of HCC or intradermal sinus tumor [8]. In addition, high AFP levels may also be found in Hodgkin’s disease, lymphoma, brain tumors, and renal cell carcinoma [9]. More importantly, some early cancer patients are usually clinically asymptomatic, leading to late diagnosis and high mortality [10,11]. Therefore, as a biomarker, AFP is of great significance for detecting some tumors in men, non-pregnant women, and children. In addition, the detection indicators of AFP in high-risk, but asymptomatic, populations are particularly important for determining early curable tumors, reducing disease-related mortality, and cost-effectiveness. The development of AFP detection technology is necessary for the early detection of diseases.

To this end, a variety of methods for detecting AFP have been developed, such as enzyme-linked immunosorbent assay [12], radioimmunoassay [13], fluorescence immunoassay [14], electrochemiluminescence [15], Raman spectroscopy [16], electrochemical immunosensor [17], and so on. Although the enzyme-linked immunosorbent assay has the advantages of simple operation, no pollution to the environment, and, specifically, it cannot provide quantitative data and only has a narrow linear range. Radioimmunoassay reagents are unstable and will decay over time, and the reproducibility of the measurement is poor. Though fluorescence immunoassay has the advantages of convenient operation, high sensitivity, and accuracy, quantitative analysis is challenging, due to the laborious labeling process [18]. Raman spectroscopy can provide high-information biomolecular signal systems and biological materials.However, traditional Raman signal spectra are too weak to be widely used in high-sensitivity quantitative and qualitative analysis. Electrochemical immunosensors, especially for label-free electrochemical immunosensors, have lots of inherent advantages, such as high sensitivity, simple operation, low cost, and easy miniaturization. However, they have the shortcomings of non-specific binding and detection limits, and they are not suitable for high-throughput analysis [19]. Therefore, the development of rapid, highly sensitive, selective, low-cost, and high-efficiency methods for the detection of AFP is essential for human disease diagnosis.

An aptamer is a single-stranded RNA or DNA molecule selected in vitro from the nucleic acid molecular library by systematic evolution of ligands by exponential enrichment (SELEX) to specifically combine targets with high affinity. The aptamer is flexible, repeatable, and easy to fix and regenerate, with no difference between batches, which has been widely used in the sensor field.Huang et al. [20] screened an AFP specifific aptamer by SELEX/micro- flfluidic chip. Dong et al. [21] selected an AFP-specifific ssDNA aptamer, named AP273, based on SELEX/capillary electrophoresis.Recently, the aptamer-based alpha-fetoprotein detection methods have been promising, and the commonly used aptamer biosensors for alpha-fetoprotein detection can be divided into two categories: optical biosensors and electrochemical biosensors. In this review, several detection methods for alpha-fetoprotein based on these two kinds of biosensors are summarized in detail.

## 2. AFP Detection Based on Optical Aptamer Biosensor

As a detection and analysis tool, the optical biosensor can not only detect many biological and chemical substances in real time, but also provide qualitative and quantitative information for a variety of biological systems. For instance, it can judge whether two molecules can interact with each other or not, and it can also calculate the equilibrium constant and kinetic constant for the formation of the complex [22]. Compared with conventional analysis technology, optical biosensors show significant advantages, including high specificity, low cost, and high sensitivity. Therefore, optical biosensors have a wide range of applications in biomedical research, pharmaceuticals, healthcare, environmental monitoring, homeland security, and battlefields, which definitely includes the detection of AFP [23,24]. In this section, several widely used AFP optical biosensing platforms, such as surface-enhanced Raman spectroscopy, dual-polarization interference, resonance light-scattering, and fluorescence and chemiluminescence biosensors, are introduced.

### 2.1. AFP Detection Based on Raman Spectroscopy

**Figure 1:**
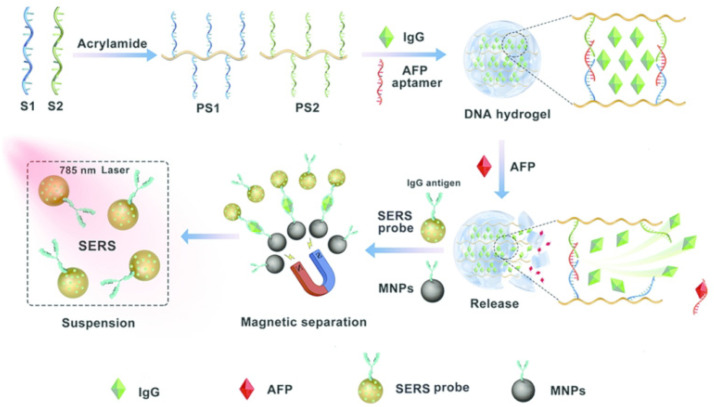
Schematic diagram of the aptamer biosensor for AFP detection based on SERS. Adapted from Ref. [30], with permission of ACS publications.

Modern analytical tools can perform highly specific identification of the samples by using only a small amount of the materials to be characterized. Raman spectroscopy, which has the advantages of high specificity, can analyze and identify materials through their specific molecular information. However, the sensitivity of Raman spectroscopy is low, which means that it cannot be used for the analysis of low-concentration samples. According to the research, a possible solution to this problem is to use metal nanostructures or particles to enhance the inherently weak Raman effect, which is called SERS (surface enhanced Raman spectroscopy) [25]. SERS combines the specificity and high sensitivity of Raman spectroscopy to analyze samples with the smallest analyte concentration. It has the advantages of simple operation, high sensitivity, fast detection speed, and good reproducibility. It is considered to be an attractive tumor marker detection and analysis technology [26,27,28]. However, most of the traditional SERS analysis is based on the reaction between the antigen and antibody of the biomarker, and expensive antibodies are required, due to the long experimental period and complicated procedures [29]. Wang et al. [30] reported a new type of SERS biosensing platform that uses a DNA hydrogel with good flexibility and stability to detect AFP with high sensitivity (Figure 1). In the absence of AFP, the pre-added IgG (immunoglobulin G) can be stably embedded in the DNA hydrogel. Thus, the SERS probe signal in the solution is not influenced, and a high signal can be maintained. After adding AFP, the aptamer chain can specifically recognize AFP and form a target-aptamer complex, which causes the rupture of hydrogel and release of a large amount of IgG. The probes conjugated to SERS and magnetically captured the antibody, and the IgG on the probes formed a sandwich structure. After magnetic separation, the probe signal on the SERS was weakened, showing a lower signal. The Raman signal, changed before and after adding AFP, can provide the quick and sensitive detection of AFP. Since the dissociation of hydrogel is directly controlled by AFP, the method can be used for quantitative detection of AFP and bringing the detection limit down to 50 pg/mL. The combination of AFP and aptamer replaced the immuneresponse of AFP and antibody, which greatly reduced the cost of the experiment. Xu et al. [31] reported self-assembled gold (AuNPs) and upconversion (Au–Au–UCNP) nanoparticle trimers, based on aptamers for the ultrasensitive detection of AFP and mucin-1. The Au–Au–UCNP trimers produced ideal optical signals, with prominent Raman enhancement and fluorescence quenching effects. The SERS intensity increased in the presence of AFP. Using the luminescence-encoded sensing system, an LOD of 0.059 aM and wide linear range of 1–100 aM for the detection of AFP were obtained. This approach has the advantage of detecting two disease biomarkers, including AFP simultaneously. The LOD in this method was lower than it was in Wang’s method.

### 2.2. AFP Detection Based on Dual-Polarization Interferometry

In recent years, tremendous attention has been paid to the interaction between aptamers and their target proteins, especially for biomarkers. Olmsted et al. [32] reported that back-scattering interferometry (BSI) was used, for the first time, to measure the binding and interaction of multiple aptamers and proteins in the solution. Dual-polarization interferometry (DPI) is a newly developed biosensor technology. It can determine the adsorption thickness and refractive index at the interface by processing the waveguide structure with alternating polarization, and it has been used to study bioaffinity interaction and evaluation of changes in protein structure [33,34]. The key advantage of DPI technology is the use of label-free reagents, which significantly simplifies the steps in the experimental procedure. In addition, the response time is shortened, which enables the data to be updated at least every 20 ms. Thus, quantitative data, such as the refractive index and thickness, can be provided in real time [35].

**Figure 2:**
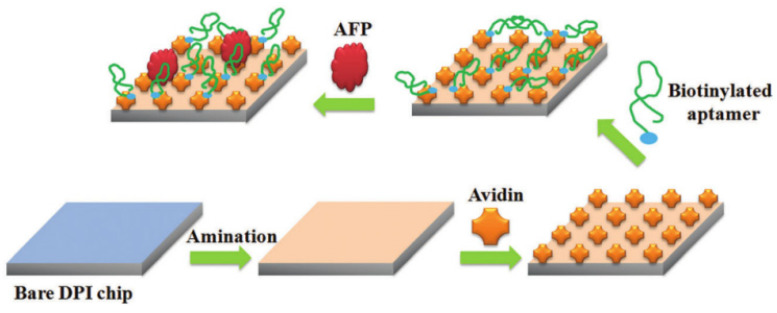
Schematic diagram of DPI-based AFP aptamer biosensor. Reproduced from Ref. [36], with permission of RSC publications (URL: https://pubs.rsc.org/en/content/articlelanding/2018/nj/c8nj04200d) (accessed on 12 August 2022).

Shao et al. [36] reported an effective method to detect and understand the interaction between protein biomarkers and aptamer DPI biosensors (Figure 2). By injecting different concentrations of AFP into the sensor modified by the aptamer, the changes in the surface quality, thickness, and density of the sensor can be monitored and recorded by the DPI online. Based on these results, the behavior and mechanism of the interaction between AFP and aptamers can be discussed. Experimental results show that two kinds of specific and non-specific bindings between the AFP and aptamer exist. AFP binds to the aptamer can change the conformation of the aptamer, which leads to the increase of the ratio of mass-to-thickness and decrease of the density in the DPI sensor. At the same time, the binding capacity and mass loading rate of high-concentration AFP and aptamer increased, indicating that the higher the concentration of AFP, the greater the possibility of being captured by the aptamer. The developed detection method proved effective for studying the interaction behaviors and mechanisms between AFP and its aptamer, and it will find wide applications in the detection and understanding of protein–aptamer interactions.

### 2.3. AFP Detection Based on Resonance Light-Scattering

**Figure 3:**
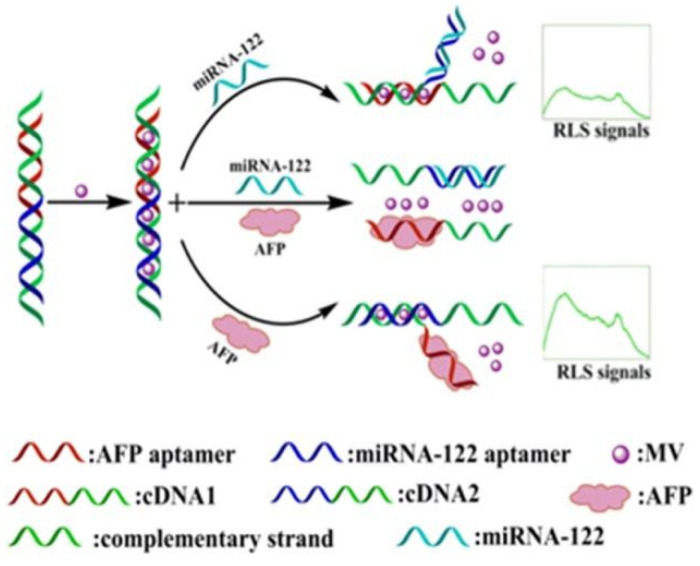
Schematic diagram of RLS-based aptamer biosensor. Reprinted from Ref. [44], with permission of Elsevier publications.

Recent studies have shown that resonance light-scattering (RLS) is a valuable technique for detecting and characterizing the extended aggregates of chromophores. When strong electronic coupling exists between chromophore units, the scattering intensity of such substances at or near the absorption wavelength will increase by several orders of magnitude [37]. Because RLS technology has high sensitivity, simplicity, and rapid performance, it has been widely used in the detection of a variety of biomolecules [38], such as DNA [39,40], protein [41], metal ions [42], and drugs [43], etc. Chen et al. [44] constructed an aptamer biosensor that uses LPS technology to detect AFP, with high sensitivity, through the electronic interaction of methyl violet (MV) and dsDNA (Figure 3). When no AFP or other target existed in the biosensor, the two DNA strands were tightly bound together. When AFP was introduced, the specific aptamer bound to the target. Thus, one side of the dsDNA was opened, and the RSL signal was changed. Under the best conditions, the LOD of the sensor was 0.94 μg/mL, and it had good selectivity and sensitivity.

### 2.4. AFP Detection Based on Fluorescence

In the development of optical biosensors based on aptamers that convert the recognition and combination of aptamers and the target to be measured into detectable signals, fluorescence detection technology provides an excellent choice for signal transduction, due to its non-destructive and highly sensitive characteristics [45], which has become one of the most commonly used methods in biosensors. Among the technologies based on fluorescence detection, especially for the fluorescence resonance energy transfer (FRET) technology, they have received extensive attention, due to the advantages of high sensitivity, low cost, and simple operation. FRET is based on non-radioactive energy transfer from the luminescent donor to the acceptor within a short distance (typically 1~10 nm), so it is widely used for quantitative analysis and the determination of biomolecules [46,47,48], small molecules [49,50], metal ions [51,52], and so on. At present, a variety of fluorescence methods based on aptamers have been developed for AFP detection. Fluorescence sensors based on FRET in nano-scale donor-acceptors, related to specific molecular recognition, have attracted widespread attention in DNA hybridization and protein interaction research, and they have also been used for the quantitative analysis of protein biomarkers.

**Figure 4:**
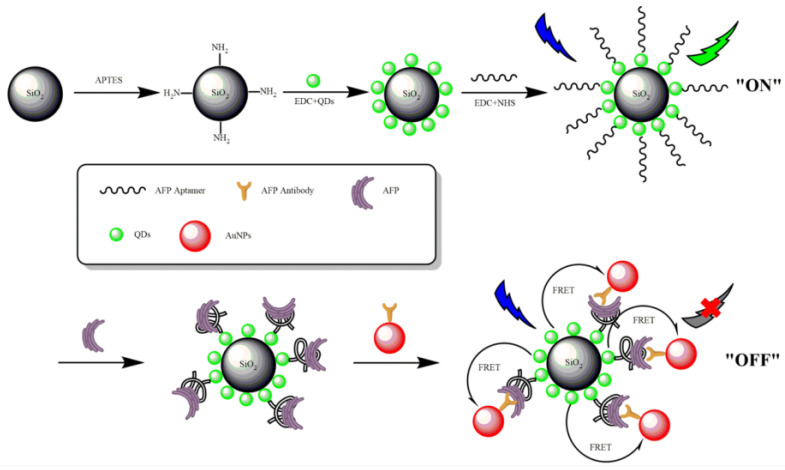
Schematic diagram of AFP sandwich aptamer biosensor based on QD fluorescence. Reproduced from Ref. [57], with permission of Elsevier publications.

Fluorescent materials, which are usually used as bioanalytical indicators in fluorescence-based biosensors, often face some obstacles in real application, due to insufficient photobleaching. With the development of nanotechnology in recent years, fluorescent nanomaterials have become high-quality and versatile fluorescent materials, owing to their strong fluorescent emission and good biocompatibility, and they are, thus, widely used in the field of fluorescent biosensors [53]. Quantum dots (QDs) have become the most commonly used energy donors to replace traditional organic dye molecules because of their unique optical characteristics, such as high quantum yield, excellent photochemical stability, size dependence, and large molarity. The extinction coefficient has wide absorption and narrow symmetrical emission spectra [54,55], and they can provide brighter, longer fluorescence lifetime, with less photobleaching effect [56]. Lu et al. [57] developed a sandwich aptamer biosensor between QDs–AuNPs conjugate pairs, based on the FRET system for AFP detection (Figure 4). First, QD was coated on SiO_2_ for agglutination, which was further covalently coupled with an AFP-specific aptamer, and an anti-AFP monoclonal antibody was coupled with acceptor gold nanoparticles (AuNPs). When QDs-labeled aptamer and AuNPs-labeled antibody were incubated with the target AFP, the specific aptamer–AFP binding caused the distance between the QDs and AuNPs to be closer to triggering FRET, which changed the fluorescence. The detection result of the fluorescent aptamer biosensor for AFP showed that the energy transfer efficiency *E* had a linear relationship with the concentration of AFP, in the range of 0.5~45 ng/mL, and the LOD was 400 pg/mL. This homogeneous aptasensor was simple, reliable, and obtained satisfying results for the detection of AFP in human serum samples.

In fluorescence-based biological analysis, energy acceptors with fluorescence quenching ability play an important role in determining the sensitivity of the analysis. A series of nanomaterials have been used as energy acceptors to build FRET-based sensing platforms, such as graphene oxide (GO) [58], palladium nanoparticles (PdNPs) [59], gold nanoparticles (AuNPs) [60], and metal organic framework (MOF) [61]. PdNPs, as a new type of nanomaterial, have a large surface area, large load of acceptor molecules, good biocompatibility, and high molar extinction coefficient. Therefore, it was frequently used as energy acceptor for FRET-based biological analysis, which has received great attention [59,62]. In addition, the nitrogen-based functional group of DNA had a strong coordination effect with PdNPs; thus, PdNPs had excellent binding ability to DNA, as well as excellent luminescence quenching ability for fluorescent dyes or fluorophores [63,64]. Li et al. [65] fabricated an aptamer nanoprobe-based biosensor that uses FRET between 5-carboxyfluorescein (FAM)-labeled AFP aptamer and PdNP to detect AFP with high sensitivity. Strong coordination occured between the nitrogen atom in the aptamer and Pd atom of PdNPs, and PdNPs can quench the fluorescence. After the addition of AFP, the green fluorescence recovered because the aptamer specifically bound to AFP, and the conformation changed. The linear relationship can be observed between the fluorescence recovery rate of FAM, and the AFP concentration with the AFP concentration range of 5~150 ng/mL, and the LOD was 1.38 ng/mL.This biosensing strategy provides a reliable and ultrasensitive protocol for the quantification of biomarkers with relevant antigens and aptamers.

### 2.5. AFP Detection Based on Chemiluminescence

Chemiluminescence (CL) was defined as the phenomenon that the product produced when a chemical reaction emits light and the exciton drops to the ground state [66]. Because of its extremely high sensitivity, it was called a powerful and important analysis technique [67]. Compared with other common spectral detection methods, CL can be detected by simple instruments, without an excitation light source and spectral analysis system. On this basis, CL had a low LOD, wide linear range, and rapid analysis performance [68]. Hu et al. [69] reported an intuitive, simple, and fast CL platform with iron-based metal-organic framework (Fe-MOF) catalysis, which used the AFP aptamers as target recognition elements to obtain the relationship between AFP concentration and chemiluminescence signal. Fe-MOF, as a catalyst with peroxidase activity, can effectively catalyze the chemiluminescence reaction between luminol and hydrogen peroxide (H_2_O_2_) and significantly enhance the CL signal. The negatively charged aptamer can bind to the positively charged Fe-MOF, thereby shielding the activity of Fe-MOF as a peroxidase and affecting the CL signal. After adding AFP to the sensor, the AFP and aptamer will interact by van der Waals force to form a special rigid structure to stimulate the generation of CL signals. Under the optimal conditions, the CL signal had a linear relationship with the concentration of AFP, in the range of 0.1 ng/L to 30 μg/L; the LOD was 77 pg/L. Wang et al. [70] used hemin@ZIF-67 composites to construct a chemiluminescence (CL) aptasensor for AFP detection. A hemin/ZIF-67 composite was prepared via covalent bonding between the carboxyl groups of hemins and cobalt ion of ZIF-67. Hemin@ZIF-67 was used as the peroxidase material, and the aptamer of AFP was modifed on its surface by electrostatic adsorptionbetween the positively charged hemin@ZIF-67 and negatively charged aptamers. Then, a simple CL aptasensor was constructed, based on the CL system. The CL signal has a linear relationship with the concentration of AFP in the range of 4 × 10^−10^ to 200 × 10^−10^ mg/mL, and the LOD was 1.3 × 10^−10^ mg/mL. The CL aptasensor has the advantages of good selectivity and high sensitivity. These two methods used MOF to increase the chemiluminescence intensity. MOF was used because of their simple synthesis process, facile operation, and low cost. However, different MOF complexes had different CL signals. Therefore, the LOD in the different methods was different.

## 3. AFP Detection Based on Electrochemical Aptamer Biosensor

The electrochemical biosensor closely combines the performance of the biosensor, that is, the high specificity in the biorecognition process and electroanalysis performance, that is, the sensitivity and structural characteristics of the electrode. According to the properties of the biological recognition elements (such as enzymes, proteins, antibodies, nucleic acids, cells, tissues, receptors, etc.), electrochemical biosensors can be roughly divided into two categories: biocatalytic equipment and affinity sensors [71,72]. Compared with other sensors, the electrochemical sensor is not highly dependent on the reaction volume, which means that it can achieve low LOD, which uses only a very small volume of the sample [73]. In addition, electrochemical detection is not affected by the fluorophores and sample components that interfere with the detection. Therefore, electrochemistry can be measured in colored or turbid samples (such as blood), without being affected by red blood cells, hemoglobin, and other components [74] in the blood, which is promising for practical application. It is precisely because electrochemical bioanalysis technology has the advantages of simple operation, wide detection range, and high selectivity that it has been widely used in clinical, environmental, industrial, and agricultural fields [75].

### 3.1. AFP Detection Based on Cyclic Voltammetry

**Figure 5:**
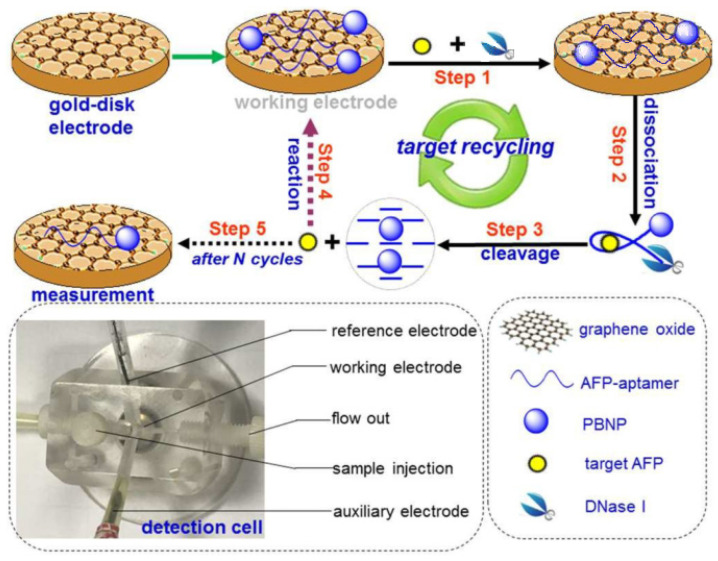
Schematic diagram of AFP detection, based on PBNPs labeled on graphene oxide. Reproduced from Ref. [83], with permission of RSC publications (URL: https://pubs.rsc.org/en/content/articlelanding/2019/an/c9an01029g) (accessed on 12 August 2022).

Voltammetry is currently one of the most commonly used electrochemical techniques, which can analyze and identify various biological analytes with high sensitivity, such as drugs, protein biomarkers, DNA damage biomarkers, and so on [76,77,78,79,80]. Voltametric biosensors can be divided into categories, i.e., cyclic voltammetry (CV), differential pulse voltammetry (DPV), linear sweep voltammetry (LSV), and square wave voltammetry (SQWV), according to different technologies [81,82].Recently, Zhang et al. [83] developed an electrochemical aptamer biosensor using Prussian blue nanoparticles (PBNPs) as signal generation tags and GO-modified materials as electrodes, which can achieve the target cyclic voltammetry to detect AFP (Figure 5). In the absence of the target AFP, the electrically active PBNPs came into contact with the electrode, due to the π-π stacking between the aptamer and nanomaterial, and the voltammetric sensor system was in the open mode. When the target was introduced into the sensor system later, the aptamer and AFP combined to form an AFP/aptamer-PBNPs complex, which made the PBNPs far away from the GO, and the voltammetric sensor system was in the off mode. In the presence of DNaseI, the complex AFP/aptamer-PBNPs were cleaved to release AFP, which recombines with the aptamer on the GO nanosheets and leads to target circulation and signal amplification. In general, the marker PBNPs would attach or separate based on whether AFP was added or not, resulting in an increase or decrease in the volt-ampere signal. The volt-ampere peak current exhibited a good linearity in the AFP concentration range of 0.01~300 ng/mL, and the LOD was 6.3 pg/mL. Importantly, this strategy provided a new horizon for the determination of disease-related proteins.

**Figure 6:**
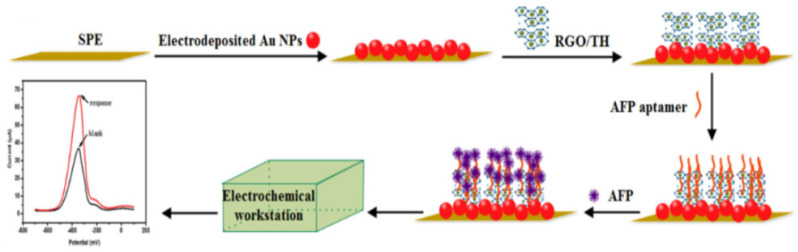
Schematic diagram of a label-free DPV aptamer biosensor based on TH/RGO/AuNPs. Reprinted from Ref. [86], with permission of Elsevier publications.

Compared with labeled sensor systems, label-free detection technology is more likely to achieve biological detection of a DNA or protein [84]. The labeling process may be time-consuming and cause high background signals. In addition, the specific recognition ability of DNA–DNA hybrids may be inhibited, to a certain extent, during the labeling process. Therefore, label-free technology can be more important for biological analysis than that of labeled biosensors [85]. Li et al. [86] used thioprotein/reduced graphene oxide/gold nanoparticles (TH/RGO/AuNPs) as a platform to capture AFP aptamers and prepared simple unlabeled DPV aptamers for AFP detection (Figure 6). The TH in the biosensor can not only effectively capture and fix AFP aptamer, due to its own amino group, but also be a signal indicator for monitoring AFP concentration. On this basis, the synergy between RGO with a high surface area ratio and AuNPs with good electrocatalytic activity can play a role in amplifying the signal and achieving the high-sensitivity detection of AFP. Therefore, after adding AFP to the system, AFP can quickly identify aptamers/TH/RGO/AuNPs and change the DPV signal of TH. Under the best conditions, the biosensor showed a linear range of 0.1 to 100.0 μg/mL, and the LOD was 0.050 μg/mL. The simple and cost-effffective sensing strategy provided a new promising platform for the design of the highly sensitive detection method, showing a potential application for the aptamer in clinical immunoassays.

### 3.2. AFP Detection Based on Electrochemical Impedance Spectroscopy

The electrochemical impedance spectroscopy (EIS) biosensor is a Faraday impedance technology and label-free bioelectronic device that measures the interaction between the analyte and receptor surface [87]. EIS can detect changes in electrical properties caused by biological recognition on the surface of modified electrodes, such as proteins, nucleic acids, microorganisms, antibodies, antigens, and cancer biomarkers [71,88,89,90]. Compared with other technologies in electrochemical sensors, EIS was less destructive to biometrics in the sensor system. It combined biometric events and signal transduction, without requiring complex and large-scale instruments, to achieve the highly sensitive detection of analytes [91,92,93,94]. Yang et al. [95] reported a label-free electrochemical biosensor for AFP determination, based on aptamer DNA/graphene oxide. By modifying GO on the electrode and carboxylating GO, it was then covalently bound to the aptamer at the amino-modified end. In the presence of AFP, the tight binding between AFP and aptamer blocked the sensor platform, resulting in blocked electron transfer, so that the signal of the EIS device changed with the change of the AFP concentration. At the same time, the CV technique was used to calculate that the sensor had a linear relationship when the AFP concentration was 0.01 to 100 ng/mL and LOD was 3 pg/mL. The proposed simple, cost-effective, and label-free strategy was promising for the determination of clinical biomarkers.

**Figure 7:**
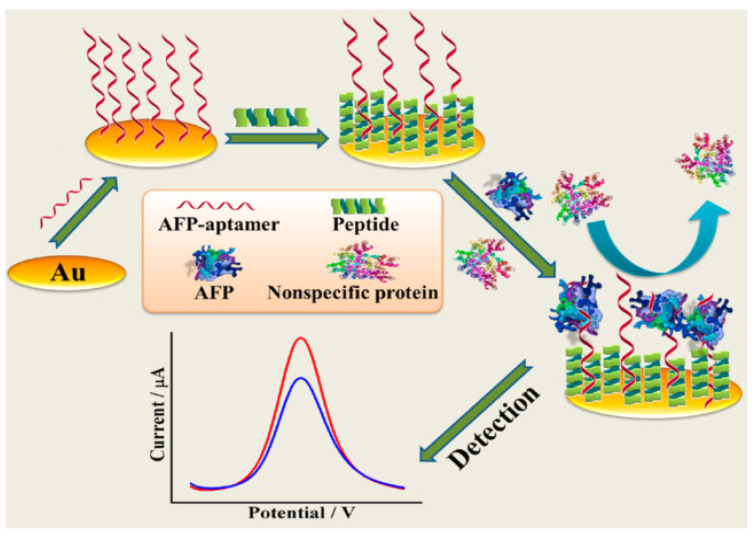
Schematic diagram of EIS detection based on aptamer DNA and zwitterionic peptide. Reproduced from Ref. [96], with permission of ACS publications.

In addition, Cui et al. [96] developed an aptamer and zwitterionic peptide, which combined the dynamic information technology of EIS analysis on modified electrodes and technology of DPV recording electrochemical response signals to realize AFP analysis with high sensitivity and low pollution detection (Figure 7). The zwitterionic peptides in the sensor platform can resist the adsorption of non-specific proteins, thus forming a dense antifouling layer that not only reduced background interference, but also ensured sufficient target binding capacity. As the AFP and aptamer bound to each other and attached to the bare gold electrode, they further hindered the charge transfer ability, thus resulting in a decrease in the DPV signal response. The dynamic response signal of EIS increased with the formation of the AFP/aptamer/peptide/Au complexes, and the resistance to electrons was significantly higher than that of bare gold electrodes. The linear range was from 10 fg/mL to 100 pg/mL, and the LOD was 3.1 fg/mL. With the high sensitivity of the electroanalytical technique, the constructed biosensor was capable of detecting the tumor biomarker AFP across its clinically relevant range with high sensitivity and low fouling. Moreover, the fabricated aptasensor exhibited promising feasibility for the quantifification analysis of AFP in real human serum samples.

### 3.3. AFP Detection Based on Giant Magnetic Impedance

A biosensor based on giant magnetic impedance (GMI) is a new type of biosensor that combines magnetism and electrochemistry. Generally, functionalized magnetic beads (MBs), magnetic fields, and the impedance of the magnetic conductor carrying the current are used as markers, external sensing platforms, and detection standards [97,98]. Compared with other devices, GMI provides great advantages for the recognition and selectivity of biomolecules, and it has excellent sensitivity (up to 600%), fast response speed, and wide frequency [99,100]. Recently, Lorenzo-Gómez et al. [101] constructed and explored the electrochemical biosensor, with affinity for AFP, based on an isothermal amplification technique for aptamers through terminal deoxynucleotidyl transferase (TdT) and rolling circle amplification (RCA). By modifying the magnetic beads in AFP and directly performing electrochemical measurement on the carbon screen printing electrode (SPCE), using different amplification techniques to perform isothermal amplification of aptamers of different lengths, the electrochemical reception signal and binding constant with AFP were improved. Both DNA amplififications improved the sensitivity and apparent binding constants from 713 nM to 189 nM for the short aptamer and from 526 nM to 32 nM for the long aptamer. The analytical sensitivity can also be improved by coupling isothermal DNA amplification strategies.

## 4. Conclusions and Outlook

In summary, the reported alpha-fetoprotein detection methods based on aptamers include: Raman spectroscopy, dual-polarization interferometry, resonance light-scattering, fluorescence, chemiluminescence, cyclic voltammetry, electrochemical impedance spectroscopy, and giant magnetic impedance. The comparison of these methods can be seen in Table 1. The optical aptamer biosensor for AFP detection has the widest linear detection range [69], and the electrochemical aptamer biosensor for AFP detection has the lowest LOD [96]. Fluorescence-based sensors are usually simpler, with a lower cost than other sensors. The results of electrochemistry-based sensors are always affected by environmental conditions. So far, the methods of AFP detection have been developed for improving in sensitivity and other aspects. The following aspects can be also improved in the future. The first one is that physical adsorption is shown in some methods. This makes some sensors have higher fluorescence background and false positive signals. The second is that the research for portable biosensorsis lacking. Although some biosensors have been miniaturized and cost-effective, they are difficult to use. Furthermore, most of sensors can detect one sample at a time. It needs to befurther developed for high-throughput detection. Thus, the optical and electrochemical types of the biosensors will continue to be developed in the future as the methods for aptamer-based alpha-fetoprotein detection that are of high sensitivity and have long-term stability, low cost, and operational convenience.

## Figures and Tables

**Table 1:** Comparison of the AFP detection methods.

Method	Analyst		Linear Range	LOD	Reference
SERS	Aptamer/nanoparticles	Aptamer/Au–Au–UCNP	1–100 aM	0.059 aM	[31]
FRET		Aptamer/QDs–AuNPs	0.5~45 ng/mL	400 pg/mL	[57]
FRET		Aptamer/PdNP	5~150 ng/mL	1.38 ng/mL	[65]
CV		Aptamer/PBNPs	0.01~300 ng/mL	6.3 pg/mL	[83]
CV		Aptamer/TH/RGO/AuNPs	0.1~100.0 μg/mL	0.050 μg/mL	[86]
CL	Aptamer/other nanomaterials	Aptamer/Fe-MOF	100 fg/mL~30 ng/mL	77 fg/mL	[69]
CL		Aptamer/hemin@ZIF-67 composites	4 × 10^−10^ to 200 × 10^−10^ mg/mL	1.3 × 10^−10^ mg/mL	[70]
RLS		Aptamer/methyl violet	-	0.94 μg/mL	[44]
SERS		Aptamer/hydrogel	50~100 ng/mL	50 pg/mL	[30]
EIS		Aptamer/graphene oxide	0.01~100 ng/mL	3 pg/mL	[95]
EIS		Aptamer/peptide/Au	10 fg/mL~100 pg/mL	3.1 fg/mL	[96]

## Data Availability

Not Applicable.
